# Reproducibility and usefulness of quantitative apparent diffusion coefficient measurements for predicting program death-ligand 1 expression in nasopharyngeal carcinoma

**DOI:** 10.1186/s40644-023-00587-2

**Published:** 2023-10-12

**Authors:** Xi Zhong, Li Li, Jinxue Yin, Yuanlin Chen, Xin Xin, Lanlan Yu, Yongfang Tang, Jiangyu Zhang, Jiansheng Li

**Affiliations:** 1 Department of Medical Imaging, Affiliated Cancer Hospital & Institute of Guangzhou Medical University, Guangzhou, 510095 China; 2 Department of Otolaryngology, The Third Affiliated Hospital of Guangzhou Medical University, Guangzhou, 510150 China; 3 Department of Pathology, Affiliated Cancer Hospital & Institute of Guangzhou Medical University, Guangzhou, 510150 China

**Keywords:** Nasopharyngeal carcinoma, Programmed death ligand 1, Magnetic resonance imaging, Apparent diffusion coefficients

## Abstract

**Background:**

Accurate assessment of programmed death-ligand 1 (PD-L1) expression status in nasopharyngeal carcinoma (NPC) before immunotherapy is crucial. We aimed to explore the reproducibility and usefulness of the quantitative apparent diffusion coefficient (ADC) measurements for predicting PD-L1expression status in NPC.

**Methods:**

We retrospectively recruited 134 NPC patients who underwent MRI scans and PD-L1 detection. A PD-L1 combined positive score (CPS) ≥ 20 was identified as high expression status. Patients were divide into two cohorts based on the MRI scanning devices, including a 1.5-T MRI cohort (n = 85, 44 PD-L1 high expression) and a 3.0-T MRI cohort (n = 49, 24 PD-L1 high expression). The mean ADC (ADC_mean_), minimum ADC (ADC_min_) and maximal ADC (ADC_max_) values were independently measured by two observers. The ADC measurement reproducibility was assessed by interclass correlation coefficients (ICC). The correlations between ADC parameters and CPS were analyzed by spearman’s correlation coefficient (r), and the performance for PD-L1expression status prediction was assessed by the area under receiver operating characteristic curve (AUC).

**Results:**

The measurement reproducibility of ADC_mean_, ADC_min_ and ADC_max_ was good in the 1.5-T MRI cohort (ICC: 0.843–0.930) and 3.0-T MRI cohort (ICC: 0.929–0.960). The ADC_mean_, ADC_min_, and ADC_max_ tended to inversely correlate with the CPS (r:-0.37 - -0.52 in the 1.5-T MRI cohort, and − 0.52 - -0.60 in the 3.0-T MRI cohort; *P* all < 0.01). The ADC_mean_, ADC_min_ and ADC_max_ yielded the AUC of 0.756 (95% CI: 0.651, 0.861), 0.689 (95% CI: 0.576, 0.802), and 0.733 (95%CI: 0.626, 0.839) in the 1.5-T MRI cohort and 0.820 (95%CI: 0.703, 0.937), 0.755 (95% CI: 0.616, 0.894), and 0.760 (95%CI: 0.627, 0.893) in the 3.0-T MRI cohort for predicting PD-L1 high expression status, respectively.

**Conclusion:**

ADC measurements may act as a reproducible and feasible method to predict PD-L1 expression status in NPC.

**Supplementary Information:**

The online version contains supplementary material available at 10.1186/s40644-023-00587-2.

## Background

Nasopharyngeal carcinoma (NPC) mortality rate was over 130,000 cases per year, and nearly 50% of NPC cases occurred in South China [1]. Approximately 10% of NPC patients developed distant metastasis at initial diagnosis, and 25% of patients developed recurrence or metastasis within 5 years after chemoradiotherapy [2]. Systemic chemotherapy has been proposed as a standard therapeutic strategy for NPC patients with recurrence or distant metastasis, but the treatment effect was frequently unsatisfactory, novel treatment strategies for advanced NPC are urgently needed [3].

Immunotherapy with checkpoint blockade inhibitors, especially programmed cell death protein-1/programmed cell death ligand 1 (PD-1/PD-L1) blockades, have shown broad application for advanced cancer therapy, such as head and neck squamous cell carcinoma (HNSCC), lung cancer, melanoma, and esophageal cancer [4–7]. For advanced NPC, clinical trial results have demonstrated that PD-1/PD-L1 blockades have significantly improved patient outcomes compared with traditional systemic chemotherapy [8, 9]. The PD-L1 status is a stratification factor for ongoing clinical trials for NPC. The immunotherapy response rate for NPC patients with high PD-L1 expression was higher than patients with low PD-L1 expression. However, the role of high PDL1 in predicting response to immunotherapy remains controversial [8–10]. The unclear predictive value of PD-L1 expression in NPC may be partly attributed to the PD-L1 detection approach. Currently, immunohistochemistry (IHC) is the most common approach for detecting PD-L1 expression in clinical practice. There are some shortcomings for PD-L1 detection in NPC, such as the tissue used for PD-L1 measurement is usually based on invasive aspiration biopsy. In addition, if the punctured NPC tissue is not enough, it may be difficult to precisely reflect the PD-L1 status due to increased tumor heterogeneity. Thus, it is important to seek an alternative quantitative method to noninvasively assess PD-L1 expression status.

Apparent diffusion coefficients (ADC) derived from diffusion-weighted imaging (DWI) could quantitatively reflect the structural and pathophysiological changes of tumors. ADC measurement is now widely applied in early diagnosis, chemoradiotherapy response evaluation and prognosis prediction in NPC [11–13]. Previous studies have demonstrated that ADC values have been significantly associated with HER-2 expression status in breast cancer, Ki-67 expression status in rectal adenocarcinoma and EGFR expression status in lung adenocarcinoma [14–16]. In addition, quantitative ADC parameters have shown potential values for predicting PD-L1 expression status in HNSCC [17] and brain metastases of lung cancer [18]. To our knowledge, the value of ADC for assessing PD-L1 expression in NPC remains unclear. Therefore, the present study aimed to explore the usefulness of ADC measurements for predicting PD-L1expression status in NPC.

## Materials and methods

### Patients

Ethical approval of the study protocol was obtained from the Ethics Review Board at Affiliated Cancer Hospital & Institute of Guangzhou Medical University, and the “waiver of informed consent” was approved by the Institutional Ethics Committee. Between January 2019 and June 2022, 213 consecutive patients confirmed with non-keratinizing differentiated or undifferentiated carcinoma (WHO classes II and III) NPC who underwent PD-L1 detection using immunohistochemistry staining were selected. Clinical features, laboratory findings, pathology and MRI data were retrospectively analyzed. The inclusion criteria were as follows: (1) nasopharyngeal MRI scan was performed within two weeks of pathological confirmation; (2) availability of complete clinical records, including patients’ age, sex, TNM stage (AJCC 8th) and clinical stage. Patients with a history of adjuvant therapy before MRI were excluded. Based on inclusion and exclusion criteria, 79 patients were excluded due to the following reasons: (1) unavailability of MRI data (n = 43); (2) incomplete clinical records (n = 15); (3) underwent chemoradiotherapy before MRI (n = 21). Finally, 134 patients (101 men, 33 women; 24 to 76 years old) were included, and patients were divide into two cohorts, including the 1.5-T MRI cohort (n = 85, patients underwent at 1.5T MRI) and the 3.0-T MRI cohort (n = 49, patients underwent at 3.0T MRI).

### PD-L1 detection and classification of expression

PD-L1 detection was performed using standard immunohistochemical (IHC) staining methods, and the PD-L1 was examined in the samples taken from the primary NPC. Biopsied tumor specimens were fixed in formalin, and paraffin-embedded tissues were sectioned at 4-µm thickness. We used the Dako Auto stained link 48 automated slide stainer (PD-L1 22c3, Dako, USA) to stain the tumor sections with PD-L1 antibody according to the recommended operator instructions. The immunostained tissue sections were scored by two independent pathologists (J.Y.Z, with 22 years and Y.L.C with 6 years of experience) who were blinded to the clinical data. The PD-L1 combined positive score (CPS) was calculated as follows: CPS = ([PD-L1 membrane staining positive tumor cells + PD-L1 membrane staining positive tumor-associated immune cells] / Total number of tumor cells) × 100.Disagreements on the assessment of CPS were resolved by consensus two weeks after the individual interpretations. PD-L1 high expression status was defined as CPS ≥ 20, and CPS < 20 was identified as low PD-L1 expression [19].

### Acquisition of MR images

A total of 85 patients underwent 1.5-T MRI (Philips Achieva) scans, and 49 patients underwent 3.0-T MRI (GE Discovery) scans. All MRI examinations were performed using a sixteen-channel head and neck coil, and the scanning range was from the skull base to the subclavian region. The imaging sequences were comprised of axial T1-weighted imaging (T1WI), axial T2-weighted imaging (T2WI), axial contrast-enhanced T1-weighted imaging (CE-T1WI), axial DWI (b = 0, 800 s/mm2), coronal fat suppression T2WI and coronal CE-T1WI. Gadolinium (Gd-DTPA; Schering, Berlin, Germany) was applied for CE-T1WI, with a bolus dose of 0.1 mmol/kg. The ADC maps were automatically generated based on the monoexponential decay model as follows: ADC = (− ln S_b_/.

S_0_) /b, where b = 800 s/mm^2^ and S_0_ was the signal intensity corresponding to no diffusion gradients (b = 0 s/mm^2^). Detailed information of the imaging parameters is shown in Table 1.

**Table 1:** MRI sequences and parameters

MRI Sequences	b value (s/mm^2^)	TR/TE (ms)	Gap (mm)	ST (mm)	FOV(cm)	Matrix	NEX
**1.5T MRI examination**							
Axial TSE T1-weighted	-	545/14	4	4	26 × 25	328 × 220	1
Axial TSE T2-weighted	-	3193/80	5	5	26 × 25	228 × 185	2
Coronal FS T2-weighted	-	3224/165	5	5	26 × 25	312 × 163	1
Axial contrast-enhanced TSE T1-weighted	-	545/14	4	4	26 × 25	252 × 219	1
Sagittal contrast-enhanced TSE T1-weighted	-	545/14	4	4	26 × 25	304 × 205	1
Axial diffusion-weighted imaging	0, 800	5500/70	2	5	26 × 25	100 × 180	2
**3.0T MRI examination**							
Axial TSE T1-weighted	-	575/20	5	5	26 × 25	256 × 192	2
Axial TSE T2-weighted	-	4000/90	5	5	26 × 25	256 × 192	2
Coronal FS T2-weighted	-	2470/68	5	5	26 × 25	256 × 256	1
Axial contrast-enhanced TSE T1-weighted	-	506/14	5	5	26 × 25	256 × 192	1
Sagittal contrast-enhanced TSE T1-weighted	-	506/14	5	5	26 × 25	256 × 192	1
Axial diffusion-weighted imaging	0, 800	6000/80	2	5	26 × 25	105 × 200	2

Abbreviations: *FS* fat suppression, *TR* repetition time, *TE* echo time, *ST* slice thickness, *FOV* field of view, *NEX* number of excitation

### ADC measurements

**Fig. 1:**
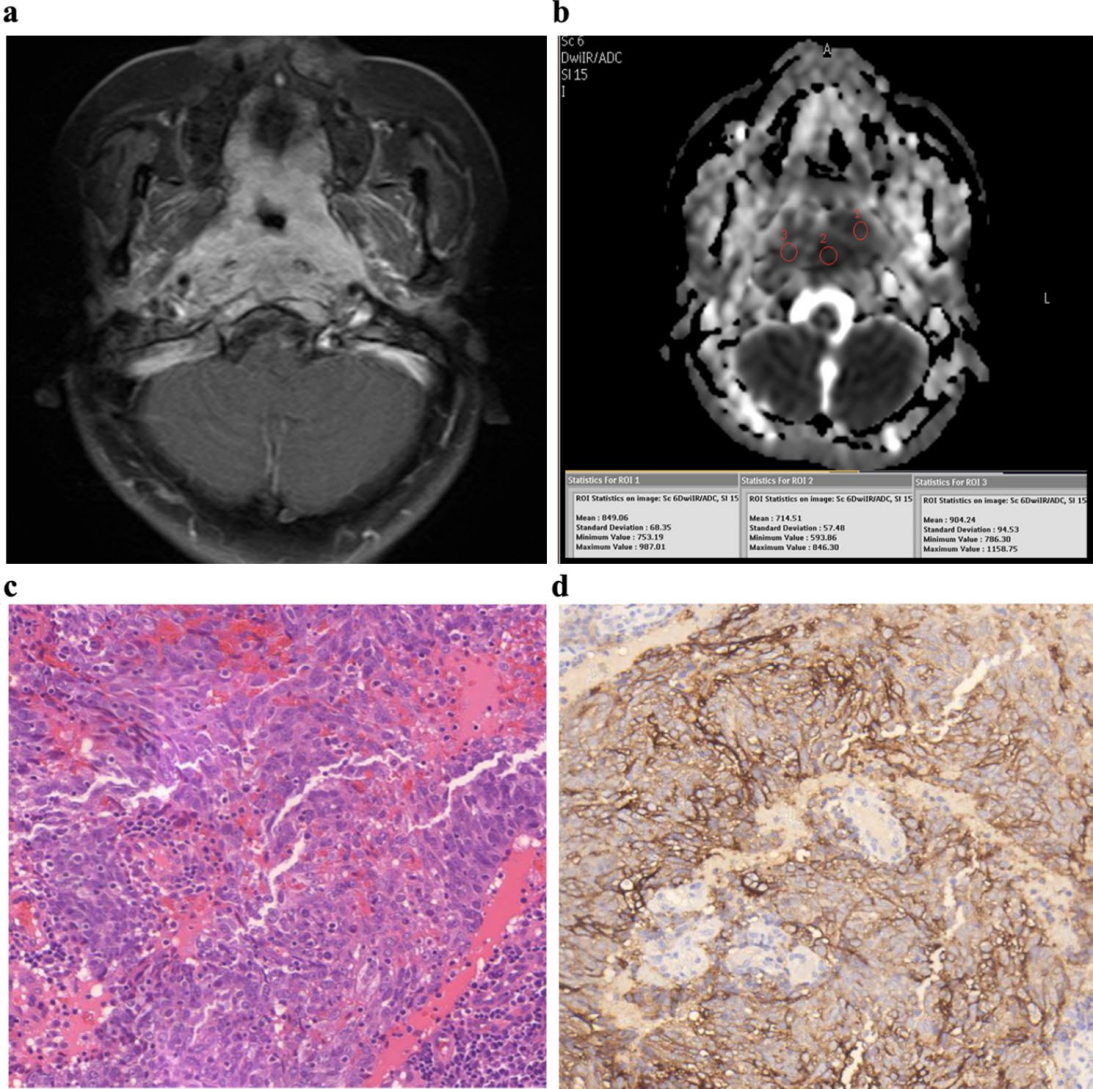
ADC measurements for a 53-year-old female with NPC. (**a**) An obvious enhancement nasopharyngeal mass with skull base bone destruction is detected on enhanced T1-w image. **(b)** Three ROIs are manually placed inside the lesion on the ADC maps; the ADC_mean_, ADC_min_ and ADC_max_ are documented. **(c)** Undifferentiated NPC is confirmed by HE staining pathology. **(d)** PD-L1 high expression status is determined by immunohistochemical (IHC) staining

The ADC measurements were independently performed on a post processing workstation by two radiologists (observer 1, X.Z with 8 years of image processing experience; observer 2, J.X.Y with 6 years of image processing experience). As shown in Fig. 1, regions of interest (ROIs) were manually positioned for each primary NPC on the section of the ADC maps that depicted the tumor maximum area to minimize the influence of a partial volume averaging effect. The regions of necrosis (obvious hyperintensity on T2-w images and unenhanced on enhanced T1-w images) were carefully avoided. As described in our previous study, three small ROIs with a similar area were drawn inside the lesion to minimize the selection bias of a single ROI measurement [20]. Then the mean ADC (ADC_mean_), minimum ADC (ADC_min_) and maximal ADC (ADC_max_) for each ROI were calculated, and the ADC parameters calculated for the three ROIs were averaged. The inter-observer reproducibility of ADC measurements between the observer 1 and observer 2 were assessed by using interclass correlation coefficients (ICC), and ADC values measured by the two observers were averaged for analysis.

### Statistical analyses

Data analyses were performed by using R statistical software (version 3.3.1, http://www.rproject.org/), and *P* < 0.05 indicated statistical significance. Categorical variables were expressed as numbers (%); quantitative variables were expressed as mean and standard deviation. An independent t-test was used to compare the differences of ADC parameters, and Pearson’s χ^2^ test or Fisher’s exact test was used for categorical variables. Spearman’s correlation coefficient (r) was used to analyze correlations between ADC parameters and CPS. ROC analyses were applied to create the optimal cut-off ADC values for predicting PD-L1 expression status, and the area under the ROC curves (AUC), sensitivity and specificity were calculated.

## Results

### Patient characteristics

Of the 134 patients enrolled in this study, 85 patients (n = 44, high PD-L1 expression; n = 41, low PD-L1 expression) that were evaluated with a 1.5-T MRI scan were assigned to the 1.5T MRI cohort, and 49 patients (n = 24, high PD-L1 expression; n = 25, low PD-L1 expression) that were examined with a 3.0-T MRI scan were assigned to the 3.0T MRI cohort. The frequency of patients with high PD-L1 expression showed no significant difference between the 1.5-T MRI cohort and 3.0-T MRI cohort (51.76% vs. 48.98%, *P* = 0.756). Detailed patient characteristics are shown in Table 2.

**Table 2:** Patients characteristics

Characteristics	1.5-T MRI cohort (n = 85)	3.0-T MRI cohort (n = 49)	*P*
**PD-L1status (No. %)**			0.756
High expression	44 (51.76%)	24 (48.98%)	
Low expression	41 (48.24%)	25 (51.02%)	
**PD-L1 CPS (mean ± SD)**	30.75 ± 26.28	37.65 ± 31.57	0.177
**Age (mean ± SD)**	50.53 ± 10.39	50.43 ± 13.36	0.961
**Gender (No. %)**			0.978
Male	64 (75.29%)	37 (75.51%)	
Female	21 (24.71%)	12 (24.49%)	
**Histology, WHO type**			0.729
II	46 (54.12%)	25 (51.02%)	
III	39 (45.88%)	24 (48.98%)	
**T stage (No. %)**			0.948
T1	7 (8.23%)	4 (8.16%)	
T2	21 (24.71%)	12 (24.49%)	
T3	38 (44.71%)	24 (48.98%)	
T4	19 (22.35%)	9 (18.37%)	
**N stage (No. %)**			0.79
N0	3 (3.53%)	2 (4.08%)	
N1	20 (23.53%)	14 (28.57%)	
N2	37 (43.53%)	17 (34.69%)	
N3	25 (29.41%)	16 (32.66%)	
**M stage**			0.783
M0	75 (88.24%)	44 (89.80%)	
M1	10 (11.76%)	5 (10.20%)	
**Clinical stage**			0.629
II	13 (15.29%)	4 (8.16%)	
III	32 (37.65%)	22 (44.90%)	
IVa	30 (35.30%)	18 (36.73%)	
IVb	10 (11.76%)	5 (10.21%)	

Abbreviations: *PD-L1* Programmed cell death ligand 1, *CPS* combined positive score

### The reproducibility of ADC measurements

The reproducibility of the ADC measurements between the two observers were very good. The ICC values of ADC_mean_, ADC_min_ and ADC_max_ were 0.930 (95% CI: 0.894, 0.954), 0.880 (95% CI: 0.821, 0.920), and 0.843 (95% CI: 0.768, 0.891) in the 1.5-T MRI cohort and 0.960 (95% CI:0.930, 0.977), 0.929 (95% CI:0.877, 0.959), and 0.931 (95% CI:0.881, 0.961) in the 3.0-T MRI cohort, respectively.

### Correlations between ADC parameters and PD-L1 combined positive score (CPS)

**Fig. 2:**
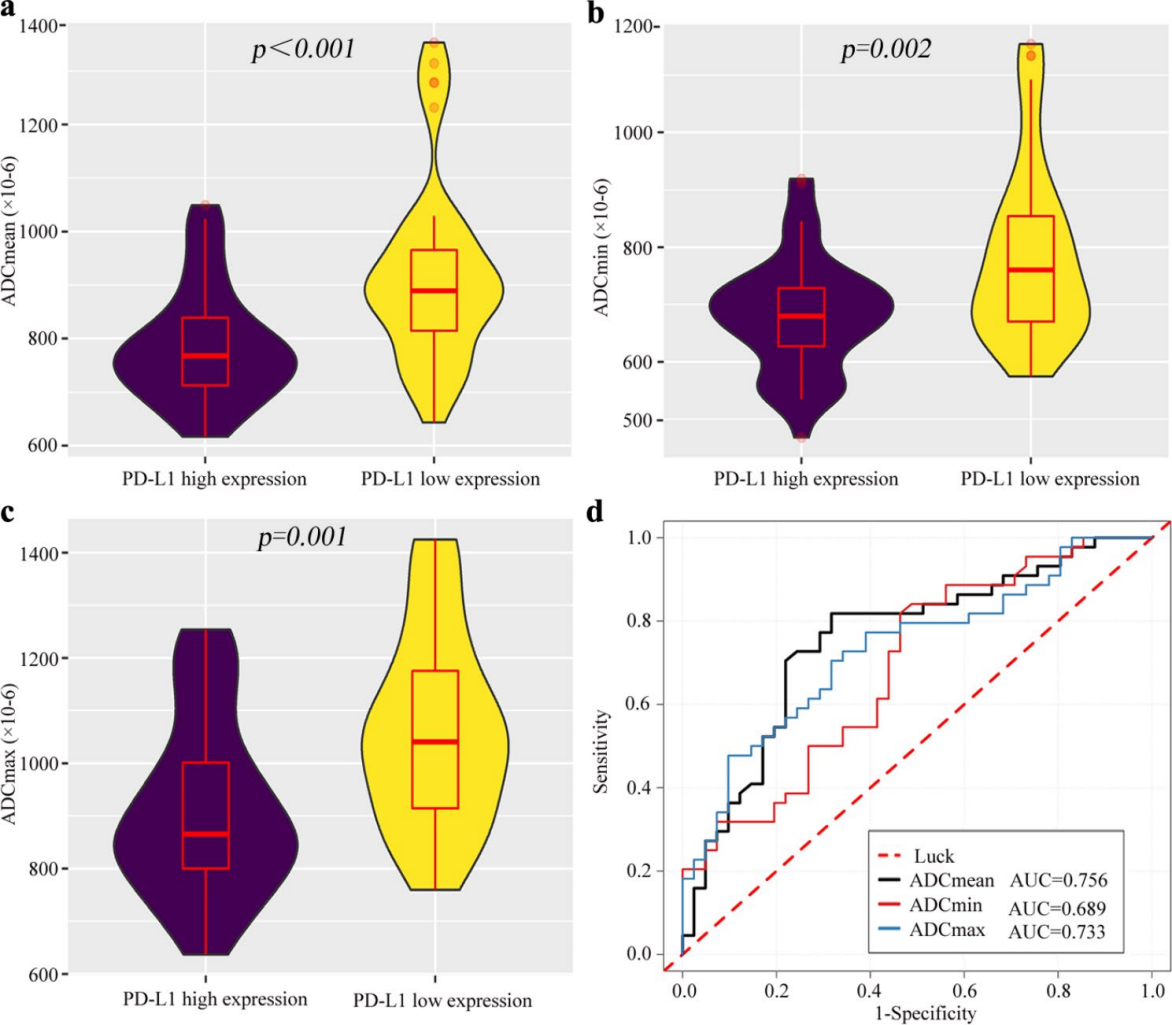
Correlations between ADC parameters and PD-L1 expression. (**a-c**) The ADC_mean_, ADC_min_ and ADC_max_ measured at 1.5-T MRI have a weak or moderate inverse correlation with the CPS, showing correlation coefficients (r) of -0.52, -0.370 and − 0.48, respectively. (**b**) The ADC_mean_, ADC_min_ and ADC_max_ measured at 3.0-T MRI have a moderate inverse correlation with the CPS, showing r of -0.60, -0.52 and − 0.58, respectively

As shown in Fig. 2, the ADC_mean_, ADC_min_ and ADC_max_ showed weak or moderate inverse correlation with the CPS (*P* all < 0.01); the correlation coefficients (r) between the ADC_mean_, ADC_min_, ADC_max_ and CPS were − 0.52, -0.37, and − 0.48 in the 1.5-T MRI cohort **(**Fig. 2a-c**)**, and − 0.60, -0.52, and − 0.58 in the 3.0-T MRI cohort **(**Fig. 2d-f**)**, respectively.

### Performances of ADC parameters for predicting PD-L1 expression status

**Fig. 3:**
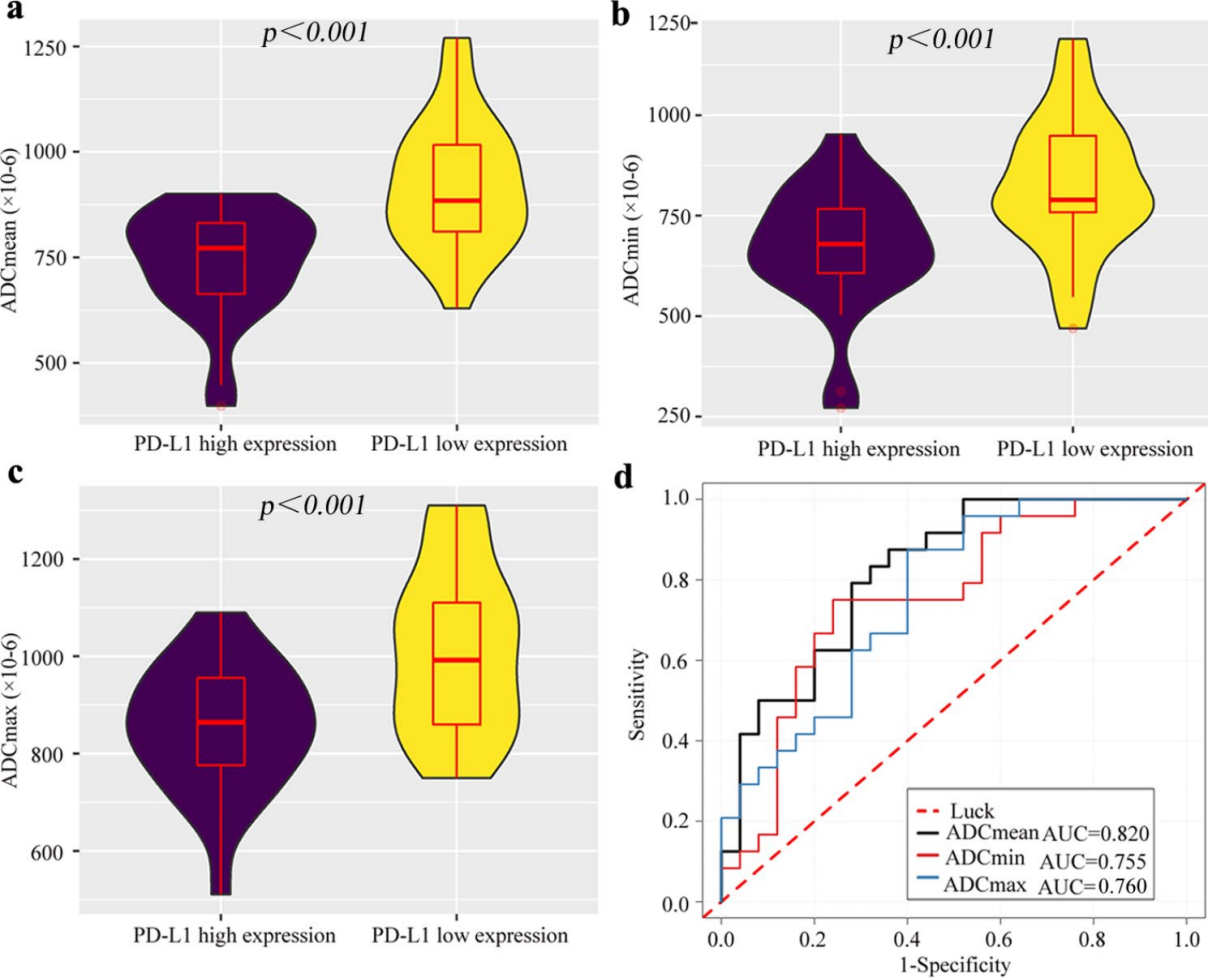
The performances of ADC parameters for predicting PD-L1 expression status in the 1.5-T MRI cohort. The ADC_mean_ (**a)**, ADC_min_ (**b)** and ADC_max_ (**c)** in PD-L1 high expression group is significantly lower than the low expression group (*P* all < 0.01). **(d)** ROC analysis of the ADC_mean_, ADC_min_ and ADC_max_ for predicting high PD-L1 expression status, with the AUC values of 0.756, 0.689 and 0.733, respectively

**Fig. 4:**
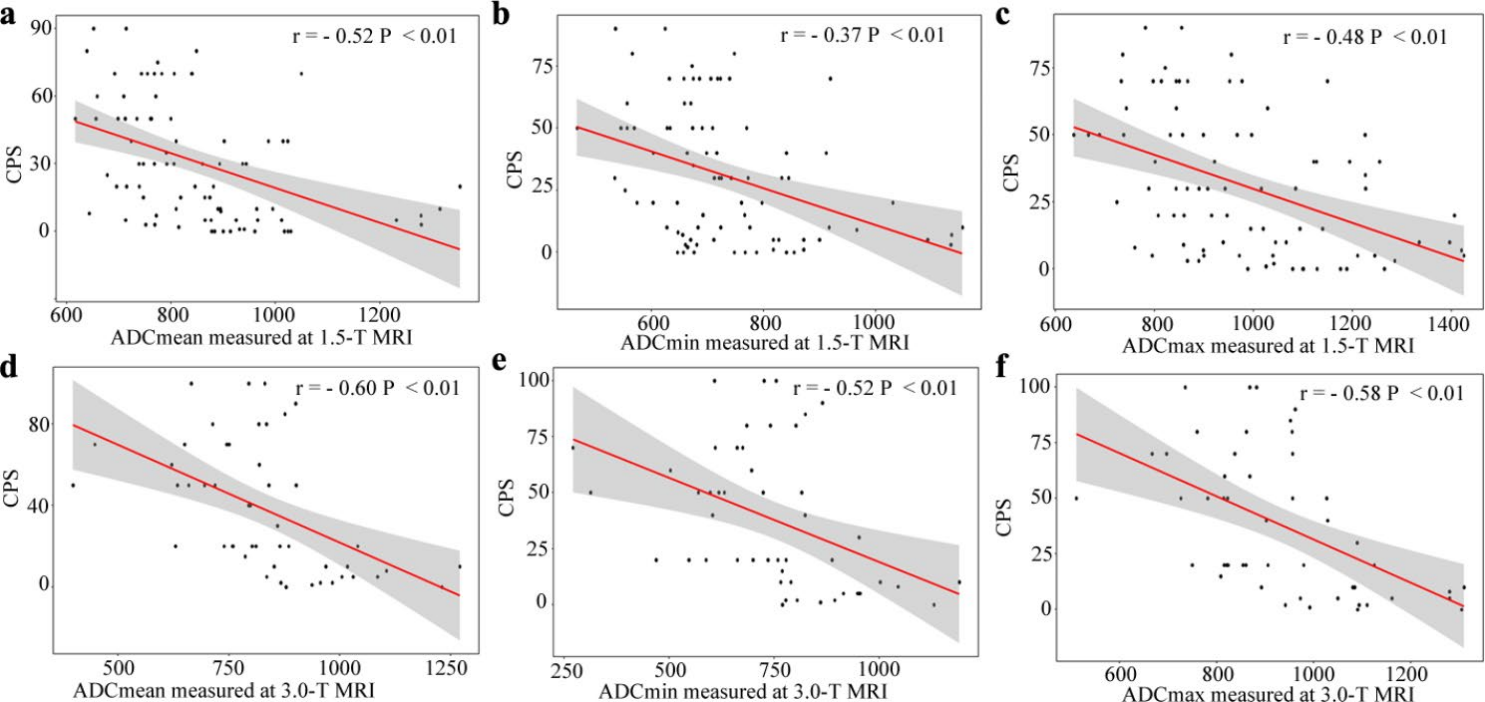
The performances of ADC parameters for predicting PD-L1 expression status in the 3.0-T MRI cohort. The ADC_mean_ (**a)**, ADC_min_ (**b)** and ADC_max_ (**c)** in PD-L1 high expression group is significantly lower than low expression group (*P* all < 0.01). **(d)** ROC analysis of the ADC_mean_, ADC_min_ and ADC_max_ for predicting high PD-L1 expression status, with AUC values of 0.820, 0.755 and 0.760, respectively

As displayed in Table 3, the ADC_mean_, ADC_min_, and ADC_max_ in PD-L1 high expression group were significantly lower than low expression group in the 1.5-T MRI cohort and 3.0-T MRI cohort (*P* all < 0.05). For predicting PD-L1 expression status, the ADC_mean_, ADC_min_ and ADC_max_ yielded the AUC values of 0.756 (95% CI: 0.651, 0.861), 0.689 (95% CI: 0.576, 0.802), and 0.733 (95% CI: 0.626, 0.839) in the 1.5-T MRI cohort **(**Figs. 3**)** and 0.820 (95% CI: 0.703, 0.937), 0.755 (95% CI : 0.616, 0.894), and 0.760 (95% CI: 0.627, 0.893) in 3.0-T MRI cohort **(**Fig. 4**)**, respectively.

**Table 3:** Difference comparisons of ADC parameters between PD-L1 high expression and low expression

ADC parameters	1.5-T MRI cohort(n = 85)			3.0-T MRI cohort (n = 49)		
	PD-L1 high expression (n = 44)	PD-L1 low expression (n = 41)	*P*	PD-L1 high expression (n = 24)	PD-L1 low expression (n = 25)	*P*
ADC_mean_ (×10^− 6^ mm^2^/s)	786.68 ± 105.69	918.63 ± 169.87	<0.001	739.96 ± 129.34	923.66 ± 153.34	<0.001
ADC_min_ (×10^− 6^ mm^2^/s)	682.31 ± 99.28	786.09 ± 152.55	0.002	669.62 ± 157.09	827.70 ± 171.01	<0.001
ADC_max_(×10^− 6^ mm^2^/s)	912.90 ± 164.69	1061.70 ± 180.89	0.001	853.17 ± 131.73	1015.40 ± 169.59	<0.001

Abbreviations: *ADC* apparent diffusion coefficient, *ADC*_*mean*_ Mean ADC, *ADC*_*min*_ Minimum ADC, *ADC*_*max*_ Maximal ADC

As displayed in Table 4, the ADC_mean_ showed an optimal efficacy for predicting PD-L1 expression status. With the cut off value of ADC_mean_ ≤ 864.00 × 10^− 6^mm^2^/s, the ADC_mean_ yielded a sensitivity of 81.82% and specificity of 68.29% for predicting PD-L1 high expression in the 1.5-T MRI cohort. With the cut off value of ADC_mean_ ≤ 876.00 × 10^− 6^mm^2^/s, the ADC_mean_ yielded a sensitivity of 87.50% and specificity of 76.00% for predicting PD-L1 high expression status in the 3.0-T MRI cohort, respectively.

**Table 4:** Performance evaluation of ADC parameters for predicting PD-L1 expression status

ADC parameters	AUC(95% CI)	Cut off value	Sensitivity	Specificity
**1.5-T MRI cohort**				
ADC_mean_ (×10^− 6^ mm^2^/s)	0.756 (0.651, 0.861)	864.00	81.82% (36/44)	68.29% (28/41)
ADC_min_ (×10^− 6^ mm^2^/s)	0.689 (0.576, 0.802)	747.00	84.09% (37/44)	53.66% (22/41)
ADC_max_ (×10^− 6^ mm^2^/s)	0.733 (0.626, 0.839)	971.00	77.27% (34/44)	68.29% (28/41)
**3.0-T MRI cohort**				
ADC_mean_ (×10^− 6^ mm^2^/s)	0.820 (0.703, 0.937)	876.00	87.50% (21/24)	76.00% (17/25)
ADC_min_ (×10^− 6^ mm^2^/s)	0.755 (0.616, 0.894)	758.00	75.00% (18/24)	76.00% (19/25)
ADC_max_ (×10^− 6^ mm^2^/s)	0.760 (0.627, 0.893)	1027.00	87.50% (21/24)	60.00% (15/25)

Abbreviations: *ADC* apparent diffusion coefficient, *ADC*_*mean*_ Mean ADC, *ADC*_*min*_ Minimum ADC, *ADC*_*max*_ Maximal ADC, *AUC* area under the ROC curve

## Discussion

In the present study, we investigated the reproducibility and usefulness of ADC measurements using 1.5-T and 3.0-T MRI for predicting PD-L1 expression status in NPC. The results indicated the reproducibility of ADC measurements were good for 1.5-T MRI and 3.0-T MRI cohort, and the ADC_mean_, ADC_min_ and ADC_max_ values tended to inversely correlate with the PD-L1 expression. In addition, we found that the ADC_mean_ showed an optimal efficacy for predicting high PD-L1 expression status, which yielded the AUC of 0.756 and 0.820 in the 1.5-T MRI and 3.0-T MRI cohort, respectively. Thus, our results indicated that ADC measurements may act as a helpful strategy to noninvasively predict PD-L1 expression status in NPC.

At present, ADC measurements have shown to be a promising strategy for early diagnosis, chemotherapy response assessment and prognosis prediction in NPC [11–13]. However, the reproducibility of ADC measurements for NPC at different MRI systems is rarely assessed. In fact, the reproducibility of ADC measurements at different MRI systems should be assessed because ADC may be influenced by field strength [21, 22]. Ye et al. assessed the ADC reproducibility of pancreas measured with different MRI systems and demonstrated that ADC measurements of the pancreas may be affected by field strength, but showed good reproducibility for both 1.5 T and 3.0T MRI systems [22]. In the present study, we found that the ICC values of the ADC measurements ranged from 0.843 to 0.930 in the 1.5-T MRI cohort and 0.929 to 0.960 in the 3.0-T MRI cohort, which suggested that the ADC measurements at different MRI systems for NPC were reproducible.

The noninvasive assessment of tumor PD-L1 expression using modern imaging techniques has produced much attention. The irregular tumor margin and peritumoral low signal intensity on hepatobiliary phase images have been independently associated with PD-L1 expression in hepatocellular carcinoma, with an AUC of 0.809 for predicting high PD-L1 expression [23]. A prediction model that consisted of functional parameters derived from PET/CT and intravoxel incoherent motion (IVIM)-MRI showed effective performance for classifying PD-L1 expression in lung cancer, with the AUC of 0.946, sensitivity of 85.29% and specificity of 91.67% [24]. As for NPC, the tumor maximum standardized uptake value (SUV_max_) measured with PET/CT was positively associated with PD-L1 expression in NPC, and yielded an AUC value of 0.733 for predicting high PD-L1 expression [25]. Thus, quantitative functional imaging may show potential value to non-invasively predict PD-L1 expression status in NPC.

In the present study, we found that ADC parameters were significantly negatively associated with PD-L1 expression in NPC. The correlation coefficients (r) between the ADC_mean_, ADC_min_, ADC_max_ and CPS ranged from − 0.37 to -0.52 in the 1.5-T MRI cohort and − 0.52 to -0.60 in the 3.0-T MRI cohort, respectively. Our findings were partly similar to the Meyer et al. study [17], in which 29 HNSCC patients who underwent 3.0-T MRI were recruited to explore the correlations between ADC values and PD-L1 expression and found that ADC values (r=-0.38) and ADC_max_ (r=-0.35) were weakly correlated with PD-L1 immune cell score. In addition, Yilmaz et al. recruited 33 patients with brain metastases of lung cancer who underwent 1.5-T MRI to determine the relationships between ADC values and PD-L1 expression indicated that the ADC_mean_ in high PD-L1 expression metastases was significantly lower than in low PD-L1 expression metastases [18]. However, the performance of ADC parameters for predicting PD-L1 expression status in previous studies had not been analyzed due to the restricted number of samples.

The strength of this study was that the performances of ADC parameters for predicting PD-L1 expression status were analyzed in both the 1.5-T MRI cohort and 3.0-T MRI cohort. The results of our study demonstrated that the ADC_mean_, ADC_min_ and ADC_max_ yielded AUC values that ranged from 0.689 to 0.756 in 1.5-T MRI cohort and 0.755 to 0.820 in 3.0-T MRI cohort for predicting PD-L1 high expression status. Furthermore, compared with ADC_min_ and ADC_max_, we found that the ADC_mean_ showed an optimal efficacy, which yielded a sensitivity of 81.82% and specificity of 68.29% in the 1.5-T MRI cohort and a sensitivity of 87.50% and specificity of 76.00% in the 3.0-T MRI cohort, respectively. Thus, our results indicated that ADC parameters, especially the ADC_mean_ may act as feasible imaging biomarkers to noninvasively predict PD-L1 expression status in NPC.

Our study has several limitations. First, the study was a single-center and retrospective study with limited sample size, and a future multicentered study with a larger sample size is needed to verify the results generated. Second, as reported in a previous study [19], we adopted CPS ≥ 20 as a cut-off value of high PD-L1 expression status. However, the optimal cut-off value of high PD-L1 expression in clinical practice for NPC remains unclear. Therefore, a multicenter, large sample prospective study should be carried out to compare the predictive performances of the different CPS cut-off values and seek the optimal and normalized cut-off value in predicting immunotherapy response. However, due to the potential relationships of ADC values with PD-L1 expression revealed in this study, the correlates of ADC values with immunotherapy response should be assessed in further studies. Third, PDL1 expression detection was based on biopsied tissue, and ADC-based testing was based on the whole solid tumor. Because of the high heterogeneity in PDL1 expression, the detection of PDL1 expression may not precisely reflect the true expression of the whole solid tumor, thus selection bias may be difficult to completely avoid in this study. Finally, the number of samples in the 3.0-T MRI cohort was obviously less than the 1.5-T MRI cohort, thus the performances of ADC parameters for predicting PD-L1 expression status between 1.5-T MRI cohort and 3.0-T MRI cohort were not compared.

## Conclusion

The ADC_mean_, ADC_min_ and ADC_max_ measurements at 1.5-T MRI and 3.0-T MRI for NPC were reproducible, and these ADC parameters showed potential value for predicting PD-L1 expression status. Thus, ADC measurements may act as a reproducible and feasible method to predict PD-L1 expression status in NPC.

### Electronic supplementary material

Below is the link to the electronic supplementary material.

Supplementary Material 1

Supplementary Material 2

## Data Availability

The datasets used and/or analyzed during the current study available from the corresponding author on reasonable request.

## Abbreviations

NPC: Nasopharyngeal carcinoma
PD-L1: Programmed cell death ligand 1
DWI: Diffusion-weighted imaging
ADC: Quantitative apparent diffusion coefficients
ADC_mean_: Mean ADC
ADC_min_: Minimum ADC
ADC_max_: Maximal ADC
ROC: Receiver operating characteristic
AUC: Area under the receiver operating characteristic curve
ICC: Interclass correlation coefficients
ROI: Regions of interest
CPS: Combined positive score
