# Supplementary material for: Reproducibility and usefulness of quantitative apparent diffusion coefficient measurements for predicting program death-ligand 1 expression in nasopharyngeal carcinoma
Source: Cancer Imaging. 2023 Oct 12;23:98. doi: 10.1186/s40644-023-00587-2 (PMC10571377; doi:10.1186/s40644-023-00587-2)
Supplement: Supplementary file 2 — Supplementary Material 2 [file 40644_2023_587_MOESM2_ESM.pdf]

06/20/2023

Editorial Certification

This document certifies that the manuscript titled "Reproducibility and usefulness of quantitative apparent diffusion coefficient measurements for predicting program death-ligand 1 expression in nasopharyngeal carcinoma" was edited for proper English language, grammar, punctuation, spelling, and overall style by one or more of the highly qualified native English speaking editors at ELIXIGEN.

Neither the research content nor the authors' intentions were altered in any way during the editing process. Documents receiving this certification should be English-ready for publication - however, the author has the ability to accept or reject our suggestions and changes. To verify the final ELIXIGEN edited version, please contact ELIXIGEN at support@elixigen.com.

*We are NOT responsible for any errors in the added content to our revised version after this date.
