# Supplementary material for: Reproducibility and usefulness of quantitative apparent diffusion coefficient measurements for predicting program death-ligand 1 expression in nasopharyngeal carcinoma
Source: Cancer Imaging. 2023 Oct 12;23:98. doi: 10.1186/s40644-023-00587-2 (PMC10571377; doi:10.1186/s40644-023-00587-2)
Supplement: Supplementary file 1 — Supplementary Material 1 [file 40644_2023_587_MOESM1_ESM.docx]

Dear editor,

Thank you for your kind letter regarding our manuscript entitled, **“Reproducibility and usefulness of quantitative apparent diffusion coefficient measurements for predicting program death-ligand 1 expression in nasopharyngeal carcinoma” (CAIG-D-23-00218),** and the referee’s remarks. We have revised and modified the manuscript in accordance with the reviewer’s comments. At this time, we have re-submitted the revised manuscript through the Author Center, and we hope to have the opportunity to publish this paper in ***Cancer Imaging.***

We have used the tracked change function of Word when revising the paper for easier reviewing purposes. All changes are marked with red text. We hope the revised manuscript will meet your standard. Below you will find our point-by-point responses to the reviewers’ comments/questions.

**Responses to Reviewer #1:**

1、It is important that point out that the PD-L1 was examined in the samples taken from the primary NPC and the ADC values were extracted from primary NPC not met nodes.

**Answer:** Thank you for your meaningful suggestion. To address the reviewer’s concern, we have emphasized that PD-L1 was examined in the samples taken from the primary NPC and the ADC values were extracted from the primary NPC in our revised manuscript (page 5, paragraph 2, line 2; page 6, paragraph 3, line 4).

2. Some spelling errors need to be corrected i.e. "weekly" or moderately inversely correlate….. in the results section.

**Answer:** Thank you very much for your valuable suggestion. To solve the grammar problems,

the native English-speaking scientists of Elixigen Company (Huntington Beach, California) edited our manuscript. In addition, we have double-checked and made English language corrections in our revised manuscript.

3. Any differences in CPS between two cohorts? If not, suggest comparing the ADC values between these two cohorts to validate whether magnetic field can influence the ADC values (supposed not, but it would be interesting to also report the results). This analysis may not the best way to investigate whether different mag filed could result in differences in ADC values, but it is still interesting to know the results (But it is not mandatory to be included in the revised version)

**Answer:** Thank you for your valuable comments. To address the reviewer’s concern, we have compared the ADC values between these two cohorts. There was no difference in the ADC values between 1.5T-MRI and 3.0T-MRI cohort. The comparison of the ADC values between the 1.5T-MRI and 3.0T-MRI cohort are shown in the following table.

**The comparison of the ADC values between the 1.5T-MRI and 3.0T-MRI cohort**

| **ADC parameters** | **3.0-T MRI cohort (n=49)** | **1.5-T MRI cohort (n=85)** | ***P* value** |
| --- | --- | --- | --- |
| ADC_mean_ (×10^-6^ mm^2^/s) | 833.68±168.47 | 850.33±154.46 | 0.562 |
| ADC_min_ (×10^-6^ mm^2^/s) | 750.28±181.16 | 732.36±137.29 | 0.520 |
| ADC_max_ (×10^-6^ mm^2^/s) | 935.96±171.50 | 984.68±187.25 | 0.137 |

Abbreviations: *ADC* apparent diffusion coefficient, *ADC_mean_* Mean ADC, *ADC_min_* Minimum ADC, *ADC_max_* Maximal ADC

4. What are the mean and median values of CPS?

**Answer:** Thank you for your valuable comment. The mean values of CPS in the 1.5T-MRI and 3.0T-MRI cohort were 30.75 ± 26.28 and 37.65 ± 31.57, respectively. The median values of CPS in the 1.5T-MRI and 3.0T-MRI cohort were 20 and 25, respectively. To address the reviewer’s concern, we have supplemented the values of CPS (mean ± standard deviation) in Table 2 in our revised manuscript.

5. Any plots to show the correlation of ADC values with CPS?

**Answer:** Thank you for your meaningful comment. We have plotted scatter diagrams (Fig. 2) to show the correlation of ADC values with CPS in our revised manuscript.

6. Any analysis involved EBV DNA? If not, suggest excluding it from the study.

**Answer:** Thank you for your valuable suggestion. We have removed EBV DNA in our revised manuscript and Table 2.

7. References format and order are not consistent. Some references in the list are missing in the content.

**Answer:** Thank you for your comments. We have made the format and order of references list consistent with the content and deleted the redundant references.

**Responses to Reviewer #2:**

1、Of note, in the introduction, the authors suggest that response rate to immunotherapy was significantly higher in high PDL1 patients in NPC, but POLARIS-02 and NCI-9742 (cited by authors) did not show statistically significant prediction of high PDL1 with IO response. To my understanding the role of high PDL1 in predicting response to immunotherapy remains unclear in NPC. With that said, both POLARIS-02 and NCI-9742 do show that high PDL1 patients numerically had higher response rates. While this does not nullify the clinical relevance of the study, it should be further discussed and acknowledged in the text.

**Answer:** Thank you for your comments and suggestions. The PD-L1 status is a stratification factor for ongoing clinical trials for NPC, and the immunotherapy response rate for NPC patients with high PD-L1 expression trended to be higher than patients with low PD-L1 expression [1-3]. However, the role of high PDL1 in predicting response to immunotherapy remains controversial. This unclear predictive value of PD-L1 expression in NPC may be partly [attributed](javascript:;) [to](javascript:;) the PD-L1 detection approach. Currently, immunohistochemistry (IHC) is the most common approach for detecting PD-L1 expression in clinical practice. There are some shortcomings for PD-L1 detection in NPC; the tissue used for PD-L1 measurement is usually based on invasive aspiration biopsy. In addition, if the punctured tissue of NPC is insufficient, it may be difficult to precisely reflect the PD-L1 status due to tumor [heterogeneity](javascript:;). Thus, it is important to seek an alternative quantitative method to noninvasively assess PD-L1 expression status. To address the reviewer’s concern, we have clarified and discussed this issue in the introduction section in our revised manuscript (page 3, paragraph 2, line 6-10; page 4, paragraph 1).

**References**

1.	Fang W, Yang Y, Ma Y, Hong S, Lin L, He X et al. Camrelizumab (SHR-1210) alone or in combination with gemcitabine plus cisplatin for nasopharyngeal carcinoma: results from two single-arm, phase 1 trials. Lancet Oncol. 2018; 19:1338-1350.

2.	Wang FH, Wei XL, Feng J, Li Q, Xu N, Hu XC, et al. Efficacy, Safety, and Correlative Biomarkers of Toripalimab in Previously Treated Recurrent or Metastatic Nasopharyngeal Carcinoma: A Phase II Clinical Trial (POLARIS-02). J Clin Oncol. 2021; 39:704-712.

3.	Ma B, Lim WT, Goh BC, Hui EP, Lo KW, Pettinger A, et al. Antitumor Activity of Nivolumab in Recurrent and Metastatic Nasopharyngeal Carcinoma: An International, Multicenter Study of the Mayo Clinic Phase 2 Consortium (NCI-9742). J Clin Oncol. 2018; 36:1412-1418.

2、The authors mention that high PDL1 in clinical practice remains unclear - it would be helpful for authors to further discuss the unclear significance of PDL1 testing in predicting immunotherapy response in nasopharyngeal cancer. There could be further contextualizing by discussing other cancers where PDL1 expression more closely correlates with IO response. There could also be discussion of possible future studies that could try to better test that ADC-based PDL1 expression correlates with response to immunotherapy (i.e. authors could describe how such prospective validation could be done).

**Answer:** Thank you for your comments. Indeed, the optimal cut-off value of PD-L1 expression in clinical practice for predicting NPC immunotherapy response remains unclear and controversial. For example, in the groups with PD-L1 combined positive score (CPS) either ≥ 20 or ≥ 1, immunotherapy or in combination with chemotherapy led to significant improvement in OS compared with the groups treated with first-line conventional therapy [1]. To clarify the real role of PD-L1 expression in predicting immunotherapy response, a multicenter, large sample prospective study should be carried out to compare the predictive performances of the different CPS cut-off values and seek the optimal cut-off value of PD-L1 expression in predicting immunotherapy response for NPC. However, due to the potential relationship between ADC value and PD-L1 expression revealed in our study, the correlation of ADC-based PDL1 expression with immunotherapy response should [be](javascript:;) [discussed](javascript:;) in further studies. A previous study indicated that ADC was a valuable biomarker for predicting induction of chemotherapy response in NPC, and noninvasive diffusion-weighted imaging was an additional indicator for guiding optical therapeutic options for patients with NPC [2]. To address the reviewer’s concern, we have clarified and discussed this issue in the discussion section in our revised manuscript (page 11, paragraph 3, lines 5-11).

**References**

[1] Saada-Bouzid E, Peyrade F, Guigay J. Immunotherapy in recurrent and or metastatic squamous cell carcinoma of the head and neck.Curr Opin Oncol. 2019;31:146–51.

[2] Chen Y, Liu X, Zheng D, Xu L, Hong L, Xu Y, et al. Diffusion-weighted magnetic resonance imaging for early response assessment of chemoradiotherapy in patients with nasopharyngeal carcinoma. Magn Reson Imaging. 2014; 32:630-637.

3、It has been posited that high heterogeneity in PDL1 expression in NPC may limit its value as a robust biomarker in this cancer setting. Perhaps the authors could also discuss this issue, and how ADC-based testing would be affected by it.

**Answer:** Thank you for your comments. In fact, the PDL1 expression detection was based on biopsied tissue and ADC-based testing was based on the whole solid tumor. Indeed, because of the high heterogeneity in PDL1 expression, the detection of PDL1 expression may not precisely reflect the true expression of the whole solid tumor, thus selection bias may be difficult to completely avoid in this study. To address the reviewer’s concern, we have clarified and added this limitation in the discussion section in our revised manuscript (page 12, paragraph 1, lines 1-4).
